# Proteasomes in Protein Homeostasis of Pluripotent Stem Cells

**Published:** 2017

**Authors:** A.V. Selenina, A.S. Tsimokha, A.N. Tomilin

**Affiliations:** Institute of Cytology, Russian Academy of Sciences, Tikhoretsky Ave. 4, Saint-Petersburg, 194064 , Russia; St-Petersburg State University, University Emb. 7/9, St-Petersburg, 199034, Russia

**Keywords:** ubiquitin-proteasome system, proteasome, immunoproteasome, embryonic stem cells, induced pluripotent stem cells

## Abstract

Embryonic stem cells (ESCs) and induced pluripotent stem cells (iPSCs) are subjects of high interest not only in basic research, but also in various applied fields, particularly, in regenerative medicine. Despite the tremendous interest to these cells, the molecular mechanisms that control protein homeostasis in these cells remain largely unknown. The ubiquitin-proteasome system (UPS) acts via post-translational protein modifications and protein degradation and, therefore, is involved in the control of virtually all cellular processes: cell cycle, self-renewal, signal transduction, transcription, translation, oxidative stress, immune response, apoptosis, etc. Therefore, studying the biological role and action mechanisms of the UPS in pluripotent cells will help to better understand the biology of cells, as well as to develop novel approaches for regenerative medicine.

## INTRODUCTION

Embryonic stem cells (ESCs) are cultured cells derived from early epiblast (primary ectoderm) cells of mammalian preimplantation embryos. ESCs can divide in culture indefinitely, avoiding the aging process and retaining their undifferentiated state and ability to differentiate into all cell – except for two extra embryonic (trophoblast and primary endoderm) – types [1, 2]. Investigation of the molecular mechanisms that control pluripotency is one of the most important pursuits in modern biology. Exploration of gene-regulatory (transcriptional) networks is an important direction in the investigation of pluripotency and exit from this cellular state through differentiation. The expression level of transcription factors, such as Oct4, c-Myc, Nanog, Klf4, and Sox2, is a critical regulatory event in the fate of pluripotent stem cells [3-6]. Even the smallest changes in the expression level of these transcription factors through interactions with other regulatory proteins can lead to differentiation or oncogenesis [4, 7-13]. Chromatin modifiers and genome stability systems also play a key role in the functioning of ESCs [14, 15]. The ability of ESCs to avoid replicative aging and, at the same time, maintain their pluripotent state is provided by the specific cellular control systems that operate in a high-intensity mode in these cells [3]. Because these are pluripotent cells of the early epiblast (natural ESC analogs) that give rise to the whole organism, including the germ line, they must possess well-functioning processes for protecting the genome from mutations. According to some studies, ESCs exhibit increased resistance to DNA damage and a low rate of genomic mutations compared to differentiated cells [16-18]. In addition, ESCs not only produce a smaller number of active oxygen forms [14, 19], but also have mechanisms to eliminate the accumulation of genotoxic and proteotoxic factors [20]. Despite the high interest to research in the field of DNA damage, regulation, and response to oxidative stress, new data demonstrate that maintenance of protein homeostasis plays one of the central roles in the functioning of ESCs [21, 22]. Protein homeostasis is a complex network of integrated and competing pathways that maintain the cellular proteome stability [23]. This network regulates all the cellular processes involved in the life cycle of proteins, including their synthesis, folding, transport, interactions, and degradation. Disruptions in protein homeostasis lead to the accumulation of damaged proteins that, in turn, negatively affect the immortality and self-renewal ability of ESCs [20]. Therefore, ESCs should obviously have a finely regulated mechanism for maintaining protein homeostasis. For example, ESCs are known to be extremely sensitive to changes in the transcription and degradation/folding of proteins [24, 25]. Some researchers argue that the loss of protein homeostasis regulation is a distinctive feature of aging; therefore, the investigation of ESCs advances our understanding of such a phenomenon as the age-related decrease in the proteome integrity [26, 27]. Due to there is some similarity between ESCs and transformed cells, a clear understanding of the protein homeostasis of ESCs may also contribute to cancer research [27].

One of the important and open questions is the generation of induced pluripotent stem cells (iPSCs) during somatic reprogramming [28, 29]. The opportunity to derive iPSCs from mouse fibroblasts by means of forced expression of key transcription factors, such as Oct4, Sox2, Klf4, and c-Myc, has substantially contributed to our understanding of the molecular mechanisms of cellular reprogramming and has opened new approaches to alternative studies that could not be implemented using model animals for a number of reasons [28, 29]. iPSCs have a morphology, proliferative capacity, and a set of endogenous pluripotency markers similar to those of ESCs and can differentiate *in vivo *and *in vitro* [30-32]. Currently, the most efficiency in reprogramming is achieved via viral delivery of reprogramming factors [28, 33-37]. Further progress in the application of this technology in research and/or medicine will depend on the opportunity to generate iPSCs in the absence of genomic modifications. Some researchers have already achieved some progress in solving this problem; for example, reprogramming with episomal vectors such as adenoviruses, transposons, purified proteins, modified RNAs, microRNAs, etc. has been demonstrated [34]. Despite the undoubted progress achieved in the generation of iPSCs, knowledge and technology are still needed in order to improve efficiency and make the reprogramming process safer and more predictable.

## THE UBIQUITIN-PROTEASOME SYSTEM

**Fig. 1:**
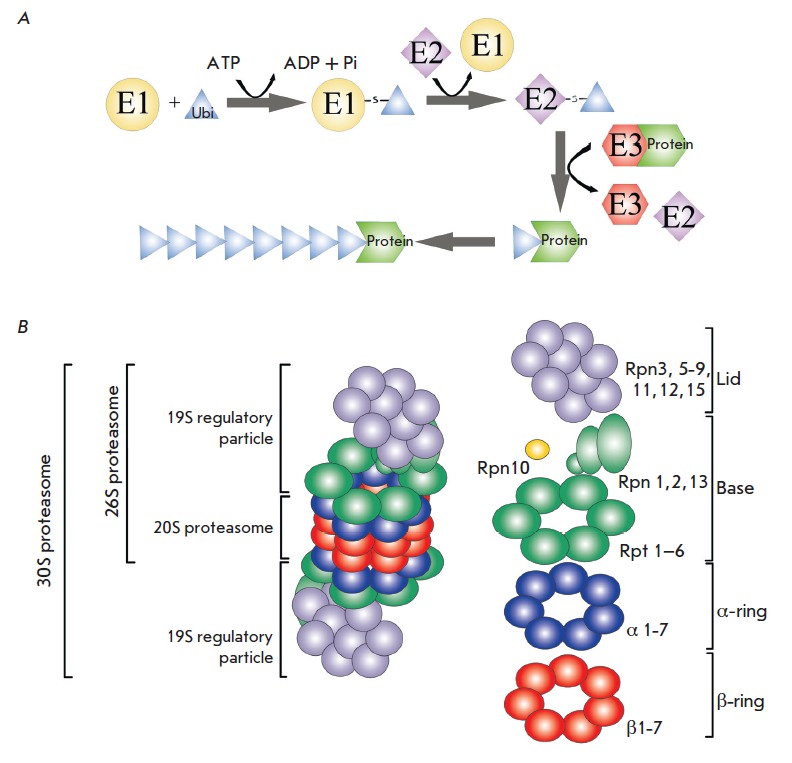
The ubiquitin-proteasome system. A – covalent binding of ubiquitin to ATP-dependent ubiquitinactivation enzymes (E1), ubiquitin-conjugating enzymes (E2), and ubiquitin ligases (E3). B – the proteasome structure (see the text for details).

**Fig. 2:**
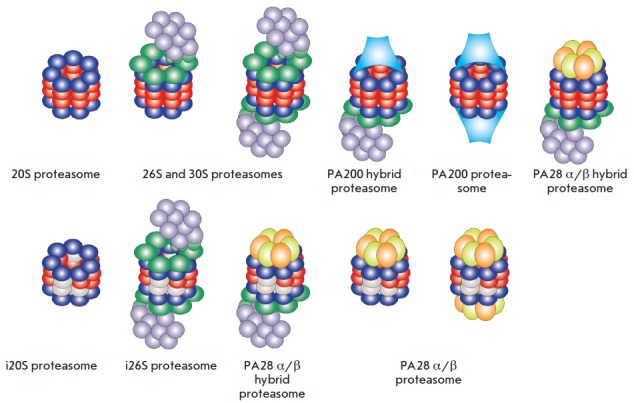
Proteasome diversity in mammalian cells. A catalytically active 20S proteasome consists of four protein rings; each ring is composed of 7 α- (dark blue) or β-subunits (7α, 7β, 7β, 7α, red). Under specific conditions, the consitutive subunits β1, β2, and β5 are replaced with the inducible subunits β1i, β2i, and β5i (light grey), which leads to the formation of immunoproteasome (i20S). The 20S proteasome can interact with one or two 19S regulatory particles (purple and green), forming the 26S or 30S proteasome, respectively; the interaction with the PA200 (blue) and PA28 regulatory particles (yellow and orange) results in hybrid proteasomes. The immunoproteasome can also bind to the 19S and PA28 regulators, forming hybrid ptoteasomes with different activities and specificities.

The ubiquitin-proteasome system (UPS) is a key participant in the maintenance of protein homeostasis. The UPS is a proteolytic apparatus of the eukaryotic cell which regulates the major cellular processes such as the cell cycle, signal transduction, transcription, translation, oxidative stress, immune response, and apoptosis [38, 39]. The UPS functions through post-translational modifications that occur via covalent attachment of ubiquitin, which is mediated by the ATP-dependent cascade of ubiquitin-activating enzymes (E1), ubiquitin-conjugating enzymes (E2), and ubiquitin ligases (E3) (*Fig. 1A*) [40, 41]. A single E1 enzyme can interact with a whole variety of E2 enzymes, and subsequent combinations between E2 and E3 provide substrate specificity and the regulation of downstream processes. Monoubiquitination is a label for signal transmission and endocytosis, while polyubiquitination leads to ATP-dependent protein degradation in the proteasome [42, 43]. The UPS is involved in the maintenance of protein homeostasis both during the cell life and in cell death; it plays an important role in both healthy and sick cells: e.g., in neurodegenerative diseases (Alzheimer’s disease), cardiac dysfunctions (transient ischemic attack), or autoimmune diseases (Sjogren’s syndrome) [44]. An important component of the UPS is a multisubunit proteolytic complex, the proteasome (*Fig. 1B*). The 20S proteasome core particle is a hollow barrel-shaped protein complex consisting of four rings, each containing seven α- or β- (7α, 7β, 7β, 7α) subunits (SUs). In eukaryotic cells, only three β-SUs have an N-terminal active-site threonine (Thr1) [45]: the SU β1/PSMB6 has a caspase-like activity; the SU β2/ PSMB7 has a trypsin-like activity; the SU β5/PSMB5 has a chymotrypsin-like activity [39, 41, 46]. The 20S core particle can interact with one or two 19S regulatory particles, forming a 26S or 30S proteasome (*Fig. 2*) [39]. The 19S regulatory complex is composed of a “base” and a “lid” subcomplexes and contains at least 18 SUs, 13 of which are ATP-independent (Rpn) SUs, and the remaining six are AAA-ATPase (Rpt) SUs [47]. The main role of the 19S lid is to recognize polyubiquitinated protein substrates using SUs Rpn10/PSMD4 and Rpn13/ADRM1 and to detach the ubiquitin molecules from them. The 19S base ensures protein unfolding, opening of the gate formed by the α-ring, and protein translocation into the catalytic cavity of the 20S proteasome [39, 47, 48]. The 20S proteasome can catalyze the degradation of proteins independent of ATP; however, like the 26S proteasome, it can interact with polyubiquitinated proteins, but the mechanisms of this process have not yet been explored [49]. The 20S particle can be activated not only by 19S particles, but also by another regulator, PA200 (*Fig. 2*) [50]. This protein also binds to the 20S proteasome, but PA200 functions and regulatory mechanisms are poorly understood. This protein is known to be mainly localized in the nucleus and able to increase proteasomal production of shorter peptides and to ensure degradation of oxidant-damaged proteins during cell adaptation to oxidative stress. In addition, PA200 expression increases in response to ionizing radiation [50]. There is another regulator of the proteasome activity, PA28 (*Fig. 2*), which is a heterohexameric or heteroheptameric complex consisting of three SUs PA28α and three SUs PA28β-PA28α3β3, or PA28α3β4, or PA28α4β3 [51]. The SU PA28 C-termini by themselves bind to the α-rings of 20S proteasome in the intersubunit pocket and, thereby, control and stabilize the open-gate conformation in the 20S proteasome, especially during the immune response [39, 52]. Under inflammatory conditions, the constitutive SUs β1/PSMB6, β2/PSMB7, and β5/PSMB5 are replaced by three inducible catalytic SUs β1i/PSMB9, β2i/PSMB10, and β5i/PSMB8. In this case, the proteasome is called an immunoproteasome (IP) (*Fig. 2*). A replacement of catalytically active SUs changes the proteasome cleavage specificity, increasing the efficiency of epitope formation for the major histocompatibility complex I (MHC I) [53-56]. Variations within the epitopes generated by IPs are caused by cleavage of proteins after basic and hydrophobic amino acid residues (trypsin- and chymotrypsin-like activities), whereas cleavage after acidic amino acid residues (caspase-like activity), according to some sources, is absent [49]. The first screening of transcriptionally active genes in human ESCs (hESCs) revealed about 900 of the most active genes, including the gene of inducible proteasomal SU β5i/PSMB8 [57]. Later, other UPS genes were found in the transcriptome hESC profile, which confirms the hypothesis on the role of UPS and protein homeostasis in maintaining ESC pluripotency [58, 59].

## PROTEASOMES IN MOUSE EMBRYONIC STEM CELLS

As mentioned above, pluripotent cells are capable of generating all cell types present in the body, which suggests the existence of rigid control over self-renewal and pluripotency. This program includes transcription factors, signaling pathways, and microRNAs closely interacting with a system of regulatory proteins and other specific proteins involved in the chromatin structure formation. This interaction forms a unique state of chromatin in pluripotent cells [60]. It is noteworthy that inhibition of the proteasome proteolytic activity or knockdown of certain proteasomal SUs in mouse ESCs (mESCs) lead to the activation of normally inactive cryptic (“hidden”) promoters [61]. The 19S complex was also shown to regulate gene expression irrespective of proteasome proteolytic activity. For example, the lid SU Rpn12/PSMD8 in mESCs controls the assembly of a transcription preinitiation complex but only in the presence of the base SU Rpt3/PSMC4 [61]. Thus, the proteasome acts as a transcriptional repressor in mESCs, preventing aberrant transcription initiation, which in turn might lead to a spontaneous exit from the pluripotency state.

The UPS actively participates in the regulation of the level and (or) functioning of various regulatory proteins in mammalian stem and germ cells, especially those proteins that are involved not only in transcription regulation, but also in the activity of signalling pathways [22]. A rapid modulation of the lifetime of these factors allows stem cells to respond to incoming signals from the environment, in response to which the cells either retain their pluripotency properties or initiate the differentiation program. The UPS is involved in the regulation of various signaling pathways: LIF/ JAK/STAT3, Nodal/TGFβ/activin, Wnt/β-catenin, Notch, and BMP. The UPS is also involved in the regulation of the activity of transcription factors, such as Rel and GATA family proteins, in various stem and progenitor cells [62-66]. It is noteworthy that all these signaling cascades are involved in the regulation of cellular pluripotency.

Proteins damaged by active oxygen forms and accumulated in mESCs have been noted to be ubiquitinated and, hence, should be further subjected to proteasome degradation [67, 68]. However, the 20S proteasome turns out to reduce a number of oxidant-damaged proteins through the ATP- and ubiquitin-independent pathways [67]. Not only 20S proteasomes, but also IPs have also been found to be involved in the degradation of oxidant-damaged proteins [69], which suggests increased expression of inducible SUs and PA28 complex proteins in mESCs. However, increased levels of β5i/ PSMB8 and PA28α/β proteins are observed only during the differentiation of mESCs [52]. Interestingly, in somatic mouse cells, such as skin fibroblasts, embryonic fibroblasts (MEFs), liver and brain cells, the level of oxidant-damaged proteins depends on the activity of IPs and hybrid PA28 proteasomes [69-71]. All these facts prove that IPs and the PA28 regulator play an important role in the degradation of oxidant-damaged proteins in somatic cells and in the differentiation of mESCs, but not in the pluripotent cells themselves.

The opportunity to generate iPSCs raised another important issue about the role of UPS in reprogramming and pluripotency induction. The pluripotency factors Oct4, Sox2, Nanog, and c-Myc, as well as Dax1, Rex1, Dnmt3l, and Msh6, have been shown to be ubiquitinated [21, 72]. Furthermore, inhibition of the proteasome activity by the reversible MG132 inhibitor causes a strong decrease in the efficiency of MEF reprogramming (our unpublished data), up to complete inhibition [21]. It is important to bear in mind that not only ubiquitination, but also phosphorylation play an important role in the maintenance of self-renewal and pluripotency by mESCs. For example, among the identified phosphorylated and ubiquitinated proteins (more than 280), many of them are somehow related to pluripotency [21]. The UPS is known to be involved in cell cycle regulation [73]. For example, ubiquitin ligase Fbw7/Fbxw7 can promote the degradation of important cell's regulators, such as c-Myc, c-Jun, cyclin- E, and Notch [74]. Interestingly, despite the fact that there is a similar level of this protein in mESCs and fibroblasts, expression of Fbw7 increases, and expression of c-Myc decreases during mESC differentiation.

In addition, knockdown of Fbw7 in mESCs causes increased expression of c-Myc, Oct4, Nanog, and Sox2 in the early differentiation stages, while inhibition of Fbxw7 expression during reprogramming increases the efficiency of iPSC generation [21]. Not only ubiquitin ligases E3, but also SUs of the 19S regulator are involved in pluripotency regulation. The deubiquitinating protein Rpn11/PSMD14 of this regulator is the key factor in maintaining pluripotency. For example, expression of Rpn11/PSMD14 decreases during the differentiation of mESCs, and knockdown of this SU in MEFs inhibits their reprogramming into iPSCs [21]. Interestingly, overexpression of Rpn11/PSMD14 in mESCs has prevented differentiation, maintaining the cells in the pluripotency state. According to our data, increased expression of the inducible proteasome SUs β5i/PSMB8 and β1i/PSMB9 occurs during reprogramming, and inhibition of the SU β5i/PSMB8 activity reduces the efficiency in iPSC generation (our unpublished data), which indicates the involvement of IPs in reprogramming.

## PROTEASOMES IN HUMAN EMBRYONIC CELLS

A microarray analysis of the transcriptome in the case of Oct4 knockdown in H1 hESCs revealed a significant change in the expression levels of 18 genes related to the UPS [75]. Inhibition of the proteasome activity in hESCs is known to lead to various consequences. For example, the reversible proteasome inhibitor MG132 affects only pluripotent stem cells, not somatic cells [24, 58, 76]. Different periods (from 20 min to 10 h) of treating with high proteasome inhibitor concentrations (20 μM MG132 and 10 μM lactacystin) failed to alter either the viability of cells or their morphology [24, 61]. Interestingly, the presence of the MG132 proteasome inhibitor, even at low doses, completely inhibited the reprogramming of MEFs into iPSCs [21] and reduced colony formation during the reprogramming of human fibroblasts, with expression of the *Oct4 *and *Nanog *genes being increased [77]. Inhibition of proteasome activity in pluripotent cells resulted in the suppression of the expression of pluripotency genes, such as Oct4, Nanog, c-Myc, Sox2, SSEA-3, Tra-1-81, and Tra-1-60, which led to the loss of self-renewal, with simultaneous activation of the expression of differentiation genes, such as FGF5 and GATA4 [24, 58, 76].

Like the mouse lid SU RPN11/PSMD14, another lid SU Rpn6/PSMD11 plays an important role in hESCs. This SU stabilizes the entire 26S proteasome complex, increasing the affinity of the 19S regulator to the 20S particle through an interaction with the SU α2/PSMA2 [24]. The Rpn6/PSMD11 expression level is high in hESCs and iPSCs, but it decreases during the differentiation of hESCs into nerve progenitor cells and mature neurons [24]. The observed decrease in the Rpn6/PSMD11 expression is accompanied by a decrease in the activity of the whole proteasome and leads to a reduced number of assembled proteasome complexes and, consequently, to the accumulation of ubiquitinated proteins in the cell. This observation, again, proves the role of the proteasome in maintaining protein homeostasis in pluripotent cells. The analysis of synthesized and functionally active proteasomes in hESCs and in comparison with nerve progenitor cells, mature neurons, fibroblasts, and hippocampal astrocytes showed the presence of a larger amount of 26S proteasomes with two 19S particles (30S proteasomes), while the amount of free 20S particles was smaller [24]. These structural rearrangements of proteasomes cause a decrease in the proteasome activity in both hESC derived cells (e.g., trophoblast) and somatic cells (e.g., fibroblasts and HEK293T cells). However, the UPS is known to play an important role in neurons, especially in the transmission of the nerve impulse [78]; so, there is still no clear explanation as to why the proteasome activity in neurons is much lower than that in hESCs.

In contrast to mESCs [52, 67], human pluripotent stem cells contain a smaller amount of oxidatively modified proteins, which is revealed when compared with human neonatal fibroblasts, as well as hESC and iPSC derivatives [79]. An increase in the number of free 20S particles during the neuronal differentiation of hESCs raises the question of whether the regulatory PA28 particle participates in this process, as it occurs in the mouse [52]. Probably, PA28 interacts with the 20S proteasome, thereby regulating its proteolytic activity. However, the emergence of the PA28 complex should be accompanied by the emergence of inducible SUs and, therefore, by the formation of IPs [69, 70]. Initially, the IP function was thought to be associated with antigen processing, protein homeostasis, and a response to oxidative stress [49, 70, 71]. Investigation of the role of IPs in maintaining hESC pluripotency demonstrated an inhibition of the proteasome chymotrypsin-like activity during the differentiation of these cells [76]. In contrast, this type of proteasome peptidase activity increases during mESC differentiation [52]. This activity is implemented by three SUs: β5/PSMB5, β1i/PSMB9, and β5i/PSMB8 [56, 80, 81]. During differentiation, the gene expression level of the constitutive proteasome SUs β1/PSMB6 and β2/PSMB7 decreases but the β5/ PSMB5 protein level remains unchanged. Despite the uncovered changes in the expression of these genes, there is no change at the protein level; at the same time, the expression of the inducible SUs β1i/PSMB9 and β5i/PSMB8 decreases both at the mRNA and protein levels [76]. These data explain the observed decrease in proteasome chymotrypsin-like activity during differentiation; however, it remains unclear if the maintenance of pluripotency is mediated by the participation of IPs. On the other hand, the use of the IP-specific inhibitors UK101 (β1i/PSMB9) and PK957 (β5i/PSMB8) activates the expression of differentiation markers and loss of hESC pluripotency [76], which indicates the role of IPs in the maintenance of pluripotency.

## CONCLUSION

**Fig. 3:**
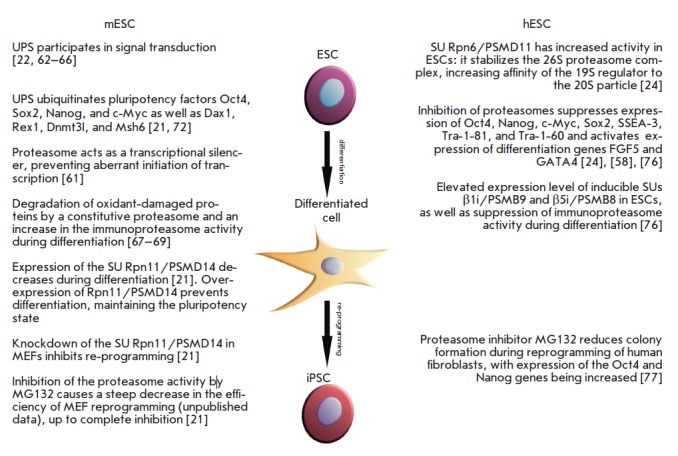
The ubiquitin-proteasome system (UPS) in mouse and human pluripotent stem cells, as well as during differentiation and pluripotency induction. Summary of the most signifciant observations regarding the role of the UPS in specified cell types and processes. References are given in square brackets.

The UPS affects the appearance and maintenance of pluripotency, as well as the loss of this state both human and mouse cells (*Fig. 3*). Proteasomes and the PA28 regulator participate in the degradation of most oxidant-damaged proteins during differentiation [52, 67], regulate the cell cycle of ESCs via E3 ligases and deubiquitinases [21], and modulate the pluripotency state through ubiquitination of the key pluripotency transcription factors, such as Oct4, Nanog, and c-Myc [21, 52]. Inhibition of proteasome activity leads to negative regulation of pluripotency factors and activation of the factors associated with cell differentiation [24, 58, 76]. In addition, IPs are also actively involved in the maintenance of protein homeostasis, cell proliferation, and differentiation, which indicates that these proteolytic complexes play an important role beyond the immune response [52, 58, 76]. Nowadays, the role of IPs in the maintenance of pluripotency and self-renewal in ESCs and iPSCs remains unclear. Further research should clarify the role of these proteolytic complexes in the induction, maintenance, and loss of pluripotency. There are also a lot of questions about the role of UPS in processes such as reprogramming and trans-differentiation, the answers to which will enable great progress in the applied fields of medicine, including regenerative medicine, substitutive cell therapy, and drug screening [29].

The strong issue today is how to increase obtain efficiency of human naive pluripotent stem cells. Some success has been achieved in this direction [82, 83]; however, it remains unknown how regulation of the UPS changes, and whether the activities of the proteasome and IP change in this process. The significance of the UPS is related to the rapid modification of cell cycle proteins, the regulation of transcription and translation, and the control of the degradation of damaged modified proteins to maintain the proliferative potential and protein homeostasis of pluripotent cells; however, a large number of the functions of the UPS in these cells remains unexplored.

ESCs and iPSCs have the unique capability of self-renewal and are pluripotent; i.e., they are able to differentiate into all cell types of three germ layers: mesoderm, endoderm, and ectoderm [2]. Mouse ESCs and iPSCs maintain pluripotency through the gene regulatory network that is based on the LIF and Wnt signaling pathways [62], while hESCs depend on the FGF and TGFβ/ Nodal/Activin signaling pathways [63]. Nowadays, the UPS is well known to be related to these signaling pathways [64-66, 84]; therefore, the discovery of new intersection nodes and mechanisms for the regulation of these pathways in the context of the UPS and pluripotent stem cells is an incredibly important and attractive prospect in biology and medicine.

## Abbreviations

UPS: ubiquitin-proteasome system
ESCs: embryonic stem cells
iPSCs: induced pluripotent stem cells
MEFs: mouse embryonic fibroblasts
SU: subunit
IP: immunoproteasome
